# CRISPR interference to evaluate modifiers of *C9ORF72*-mediated toxicity in FTD

**DOI:** 10.3389/fcell.2023.1251551

**Published:** 2023-08-07

**Authors:** Sarah Pickles, Desiree Zanetti Alepuz, Yuka Koike, Mei Yue, Jimei Tong, Pinghu Liu, Yugui Zhou, Karen Jansen-West, Lillian M. Daughrity, Yuping Song, Michael DeTure, Björn Oskarsson, Neill R. Graff-Radford, Bradley F. Boeve, Ronald C. Petersen, Keith A. Josephs, Dennis W. Dickson, Michael E. Ward, Lijin Dong, Mercedes Prudencio, Casey N. Cook, Leonard Petrucelli

**Affiliations:** ^1^ Department of Neuroscience, Mayo Clinic, Jacksonville, FL, United States; ^2^ Neuroscience Graduate Program, Mayo Graduate School, Mayo Clinic, Jacksonville, FL, United States; ^3^ Genetic Engineering Core, National Eye Institute, National Institutes of Health, Bethesda, MD, United States; ^4^ Department of Neurology, Mayo Clinic, Jacksonville, FL, United States; ^5^ Department of Neurology, Mayo Clinic, Rochester, MN, United States; ^6^ National Institute of Neurological Disorders and Stroke, National Institutes of Health, Bethesda, MD, United States

**Keywords:** frontotemporal dementia, amyotrophic lateral sclerosis, STMN2, *C9orf72*, CRISPR interference, TDP-43

## Abstract

Treatments for neurodegenerative disease, including Frontotemporal dementia (FTD) and Amyotrophic lateral sclerosis (ALS), remain rather limited, underscoring the need for greater mechanistic insight and disease-relevant models. Our ability to develop novel disease models of genetic risk factors, disease modifiers, and other FTD/ALS-relevant targets is impeded by the significant amount of time and capital required to develop conventional knockout and transgenic mice. To overcome these limitations, we have generated a novel CRISPRi interference (CRISPRi) knockin mouse. CRISPRi uses a catalytically dead form of Cas9, fused to a transcriptional repressor to knockdown protein expression, following the introduction of single guide RNA against the gene of interest. To validate the utility of this model we have selected the TAR DNA binding protein (TDP-43) splicing target, stathmin-2 (*STMN2*). *STMN2* RNA is downregulated in FTD/ALS due to loss of TDP-43 activity and STMN2 loss is suggested to play a role in ALS pathogenesis. The involvement of STMN2 loss of function in FTD has yet to be determined. We find that STMN2 protein levels in familial FTD cases are significantly reduced compared to controls, supporting that STMN2 depletion may be involved in the pathogenesis of FTD. Here, we provide proof-of-concept that we can simultaneously knock down Stmn2 and express the expanded repeat in the Chromosome 9 open reading frame 72 (*C9ORF72*) gene, successfully replicating features of C9-associated pathology. Of interest, depletion of Stmn2 had no effect on expression or deposition of dipeptide repeat proteins (DPRs), but significantly decreased the number of phosphorylated Tdp-43 (pTdp-43) inclusions. We submit that our novel CRISPRi mouse provides a versatile and rapid method to silence gene expression *in vivo* and propose this model will be useful to understand gene function in isolation or in the context of other neurodegenerative disease models.

## Introduction

Individuals diagnosed with FTD experience impaired behavioral and language function due to neuronal loss in the frontal and temporal lobes of the brain, whereas those with ALS will suffer a progressive loss of motor neurons in the cortex, brain stem, and spinal cord, resulting in paralysis and eventually death. At autopsy, almost all ALS cases and roughly half of FTD cases show a mislocalization of TDP-43 from a diffuse nuclear distribution to nuclear and cytoplasmic inclusions in affected brain regions (Ling et al., 2013). Individuals with an expanded G_4_C_2_ repeat in the *C9ORF72* gene, the most common genetic cause of both FTD and ALS (C9FTD/ALS), have TDP-43 positive inclusions, as well as C9FTD/ALS specific pathology related to the expanded repeat. In particular, repeat-containing sense (G_4_C_2_) and antisense (C_4_G_2_) RNA transcripts lead to the formation of nuclear RNA foci and accumulation of five dipeptide repeat proteins produced through unconventional repeat-associated non-AUG (RAN) translation, including poly(GA), poly(GP), poly(GR), poly(PR) and poly(PA) (Balendra and Isaacs, 2018). Along with the *C9ORF72* repeat expansion, pathogenic mutations in the Progranulin (*GRN*) gene are also causative for FTD with TDP-43 pathology, leading to haploinsufficiency of the encoded progranulin protein (Rhinn et al., 2022). At present, those diagnosed with either FTD or ALS have limited treatment options, strongly underscoring the need for better insight into disease mechanisms to facilitate the discovery of more effective therapeutic strategies (Andrews et al., 2020).

Currently, the field’s ability to evaluate the role of newly identified genetic risk factors, disease modifiers, and therapeutic targets in pre-clinical animals is hampered by the significant time and costs associated with developing conventional knockout or transgenic mouse models. Genome editing in the mouse central nervous system (CNS) has been achieved by developing CRISPR/Cas9 knockin mice and delivery of single guide RNA (sgRNA) via adeno-associated virus (AAV) (Platt et al., 2014). These mice provide a model to probe the loss of function of virtually any gene. Epigenetic modulation of gene expression via CRISPR activation (CRISPRa) or interference (CRISPRi) has recently been described, whereby a catalytically dead form of Cas9 is fused to transcriptional enhancer or repressor and targeted to the transcription start site of a gene of interest by sgRNA, leading to histone acetylation or methylation and corresponding changes in gene expression (Kampmann, 2018). CRISPRa/i has been successfully used to alter gene expression in the mouse brain (Zheng et al., 2018; Carpenter et al., 2020; Gemberling et al., 2021). Compared to Cas9, CRISPRi avoids tissue mosaicism due to incomplete loss of function and gain of function phenotypes within cell populations (Smits et al., 2019) and does not induce double-strand breaks, mitigating DNA-damage associated toxicity and large genomic deletions or translocations (Haapaniemi et al., 2018; Ihry et al., 2018; Kosicki et al., 2018). CRISPRi mice may be particularly advantageous when combined with established mouse models of FTD/ALS to avoid complex breeding strategies which may be labor-intensive, costly, and require multiple crosses over several generations.

STMN2 is a microtubule-binding protein that is highly expressed in neurons and plays an important role in axonal growth and maintenance (Shin et al., 2012; Shin et al., 2014). *STMN2* splicing is regulated by TDP-43, such that in conditions of TDP-43 deficiency, a cryptic exon is included within the RNA, leading to the production of a non-functional truncated *STMN2* variant and the concomitant loss of full-length *STMN2* in ALS and FTD cases (Klim et al., 2019; Melamed et al., 2019; Prudencio et al., 2020). Flies lacking the *STMN2* ortholog have defective synapse stability at the neuromuscular junction (NMJ) (Graf et al., 2011), accompanied by paralysis and reduced life span (Duncan et al., 2013). Knockdown of *Stmn2* in the midbrain of mice results in the selective loss of dopaminergic neurons and impaired motor function, pointing towards a role for Stmn2 in neuronal survival (Wang et al., 2019). Collectively, Stmn2 knockout mice exhibit deficits in motor behavior due to the denervation of the NMJ (Guerra San Juan et al., 2022; Krus et al., 2022; Lopez-Erauskin et al., 2022). Moreover, motor deficits were rescued by crossing Stmn2 knockout mice to mice expressing human STMN2, suggesting that human STMN2 performs a similar role in NMJ maintenance (Guerra San Juan et al., 2022). Similarly, restoring STMN2 levels in induced pluripotent stem cell (iPSC)-derived motor neurons depleted of either STMN2 or TDP-43, rescued axonal regeneration and growth defects (Klim et al., 2019; Melamed et al., 2019). Taken together, these data strongly support that loss of STMN2 protein contributes to ALS pathogenesis. However, as *STMN2* RNA levels are also reduced in the frontal cortex of FTD patients (Prudencio et al., 2020), it is important to assess the contribution of STMN2 deficiency to FTD-associated phenotypes, in particular given the recent development of therapeutic strategies to rescue *STMN2* splicing and restore STMN2 expression and function. In addition, given that the TDP-43 binding site within intron 1 of the human *STMN2* gene is not conserved in mice (Melamed et al., 2019; Klim et al., 2021), previous mouse models with impaired Tdp-43 function lacked Stmn2 misprocessing and loss of function. To this end, the current goals of the study include: 1) Assess whether STMN2 loss of function is a feature of FTD, 2) generate a CRISPRi mouse model using Stmn2 depletion as a proof-of concept and 3) combine CRISPRi of Stmn2 with a *C9ORF72* expanded repeat. The creation of this dual model serves to demonstrate the ease with which knockdown can be combined with established neurodegenerative disease models and will allow us to evaluate if Stmn2 deficiency in the presence of the expanded *C9ORF72* repeat and endogenous Tdp-43 pathology exacerbates pathology, thereby providing a more complete model of FTD.

## Materials and methods

### Human post-mortem tissues

The Mayo Clinic Florida Brain Bank provided post-mortem brain tissue from individuals with confirmed FTD with TDP-43 inclusions and individuals without neuropathological features. We obtained written informed consent from all participants or their family members. The Mayo Clinic Institutional Review Board and Ethics Committee approved all protocols associated with the collection of human tissues. The sample size was determined based on the availability of tissue. A description of patient demographic information for both control individuals and FTD cases can be found in Supplementary Table S1.

### Animal studies

All animal procedures were conducted in accordance with the National Institutes of Health Guide for Care and Use of Experimental Animals and were approved by both the Mayo Clinic Institutional Animal Care and Use Committee (IACUC Protocol number A00005405-20) and the National Eye Institute ACUS committee (ASP number NEI-626).

### Generation of CRISPRi knockin mouse model

The CRISPRi knockin mouse line used in this study was created at the Genetic Engineering Core, National Eye Institute, National Institutes of Health. The system uses a human codon-optimized dCas9 from *Streptococcus* pyogenes, fused with a nuclear localization sequence (NLS, 2X), an HA tag, and the coding sequence of blue fluorescent protein (BFP). The modified dCas9 is fused with the KRAB (Krüppel-associated box) domain of the human *KOX1* gene at its C-terminus, which facilitates the recruitment of chromatin-modifying factors to the targeted gene locus (Margolin et al., 1994).

To create the CRISPRi knockin mouse line, a vector was designed to target the Rosa26 locus of the previously constructed mouse genome (Liu et al., 2019). The CRISPRi cassette containing the essential elements of the system was obtained by PCR amplification from pHF-SFFV-dCas9-BFP-KRAB, a gift from Stanley Qi and Jonathan Weissman (Addgene plasmid #46911) (Gilbert et al., 2014). The cassette was then inserted between the Cytomegalovirus early enhancer/chicken β actin (CAG) promoter and the rabbit beta-globin polyadenylation (RBG-PA) signal sequence in the mouse Rosa26 targeting vector, which contains a phosphoglycerate kinase-neomycin (PGK-Neo) cassette flanked by two FRT sites. To ensure insulation of the transgene, the CAG-CRISPRi-RBG-PA cassette was flanked by two copies of the 5′-HS4 chicken β-globin (cHS4) insulator (Chung et al., 1993).

To introduce the CRISPRi knockin allele at the Rosa26 locus, the targeting vector was linearized using AscI and then electroporated into mouse W4 embryonic stem (ES) cells as described (Bradley et al., 1984). Homologous recombination (HR) events were selected using neomycin (neo) selection followed by HR screening using a long genomic PCR assay. Two PCR assays were performed: one with forward primer 5′-CGG​CAC​TAC​TGT​GTT​GGC​GGA​CTG​GCG​GGA​C-3′ and reverse primer 5′-GAA​CAT​ACG​TCA​TTA​TTG​ACG​TCA​ATG-3′ to detect the left arm HR (2.85 kb PCR product) and another with forward primer 5′-GGT​GTG​GCG​GAC​CGC​TAT​CAG​GAC​ATA​GC-3′ and reverse primer 5′-CAT​GTT​TAG​TAA​TGG​CTG​CAG​ACT-3″ to detect the right arm HR (2.145 kb PCR product). ES clones carrying the CRISPRi knockin allele at the Rosa26 locus were then expanded and microinjected into blastocyst embryos obtained from C57BL6/J female donors. Male chimeric F0 pups with 90% or higher agouti coat color were crossed with wildtype C57Bl6/J females to achieve germline transmission of the knockin allele. F1 pups carrying the germline-transmitted knockin allele were bred with wild-type C57Bl6/J mice for several generations until they became congenic with the C57Bl6/J background. Finally, the neo cassette was removed by crossing the CRISPRi knockin mice with a germline flippase (FLP) recombination strain (actin-FLP, Jackson lab).

### Generation of AAV vectors and P0 injections

To produce AAV expressing sgRNA and sgRNA plus a *C9ORF72* G_4_G_2_ repeat, the coding sequence for the mouse U6 promoter, including modified stem loop 1, 2 and guide sequence site from pU6-sgRNA EF1Alpha-puro-T2A-BFP, a gift from Jonathan Weismann and Martin Kampmann (Addgene plasmid #60955) (Gilbert et al., 2014), were cloned into the pAAV CBA EGFP backbone and pAAV CBA *C9ORF72* with 2 or 149 G_4_C_2_ repeats (referred to as 2R and 149R, respectively) (Chew et al., 2015). To generate AAV sgStmn2 we selected four sgRNA sequences against Stmn2, as described previously (Horlbeck et al., 2016). Top and bottom oligonucleotides were annealed and ligated into BstXI and BlpI restriction sites within the AAV sgRNA or sgRNA-2R/149R backbone. Sequences for sgRNA are as follow sgStmn2#1: 5′-GGC​CGC​GGA​GAG​AGC​GAG​AG-3′, sgStmn2#2: 5′-GGG​AGT​GGT​GGC​GAA​GGC​AA-3′, sgStmn2#3: 5′-GGG​GAT​GTG​CAC​GCA​CGG​AG-3′, sgStmn2#4: 5′-GCG​AGA​GAG​GAG​TCT​GAG​TA-3′ and sgCtrl: 5′-GGACTAAGCGCAAGCACCTA-3′.

To produce AAV9 virus we followed a previously described protocol (Chew et al., 2015). Briefly, AAV vectors were co-transfected with helper plasmids in HEK293T cells. After 72 h, cells were harvested, treated with 50 Units/mL Benzonase (Sigma-Aldrich), and lysed by several cycles of freeze-thaw. The virus was purified using a discontinuous iodixanol gradient and diluted in PBS.

Intracerebroventricular injections (ICV) of AAV were performed as previously described with some minor modifications (Chew et al., 2015). In a few words, a 30-gauge needle attached to a 10 μL syringe (Hamilton Company) was inserted into the lateral ventricle of cryo anesthetized CRISPRi pups at postnatal day 0 (P0). Two microliters (1.5E10 genomes/μL) of AAV9 virus were manually injected into each ventricle. Following injection, pups were placed on a heated pad until they recovered from anesthesia, at which time they were placed back into their home cage. Approximately three-to-four-week later pups were weaned, DNA was extracted from tails and genotyped for the presence of CRISPRi transgene using the following primer set: dCas9 forward: 5′- ATC​AAC​CGG​CTG​TCC​GAC​TA-3′, dCas9 reverse: 5′-CCT​CTG​GGT​AAT​CAG​CTT​GG-3′.

### Mouse tissue processing

Mice were euthanized by CO_2_ and brains were harvested and cut sagittally across the midline. Sagittal half brains were fixed in 10% formalin, embedded in paraffin, sectioned, and mounted on glass slides. The other half brain was dissected and frozen on dry ice. Frozen mouse forebrain tissues were homogenized in ice-cold Tris-EDTA (TE; 50 mM Tris, pH 8.0, 50 mM NaCl, 1 mM EDTA), with 2× protease and phosphatase inhibitors.

### RNA extraction, cDNA synthesis, and qRT-PCR

To extract total RNA, brain homogenates were mixed with Trizol LS (Thermo Fisher Scientific) and frozen. The following day, RNA was extracted using the Direct-zol RNA MiniPrep kit (Zymo Research). cDNA was synthesized from 250 ng of RNA using the High-Capacity cDNA transcription kit (Applied Biosystems). Quantitative reverse transcription-PCR (qRT-PCR) was performed using the SYBR GreenER qPCR SuperMix (Invitrogen, Thermo Fisher Scientific). Each sample was run in triplicate and processed on a Prism 7900HT Fast Real-Time system (Applied Biosystems). Samples were analyzed using the ΔΔCt method and normalized to the endogenous controls (*Rplp0* and *Gapdh*). The following primers and their sequences were used:

*Stmn1* forward: 5′-TCT​GTC​CCC​GAT​TTC​CCC​C-3′, *Stmn1* reverse: 5′-AGC​TGC​TTC​AAG​ACT​TCC​GC-3’.

*Stmn2* forward: 5′-GCA​ATG​GCC​TAC​AAG​GAA​AA-3′, *Stmn2* reverse: 5′-GGT​GGC​TTC​AAG​ATC​AGC​TC-3′.

*Stmn3* forward: 5′-CCA​GCA​CCG​TAT​CTG​CCT​AC-3′, *Stmn3* reverse 5′- CAG​AGG​CCC​GCT​TAT​CCA​G-3’.

*Stmn4* forward: 5′-GCG​TCA​TCT​CTG​ATA​TGG​AAG​TC-3′, *Stmn4* reverse: 5′- CTC​TTC​TAA​TGA​TGG​GTC​TCG​C-3’.

*Gapdh* forward: 5′-CATGGCCTTCCGTGTTCCTA-3′, *Gapdh* reverse: 5′-CCTGCTTCACCACCTTCTTGAT-3′.

*Rplp0* forward: 5′-ACTGGTCTAGGACCCGAGAAG-3′, *Rplp0* reverse: 5′-CTCCCACCTTGTCTCCAGTC-3′.

*Iba1/Aif1* forward: 5′-GGA​TTT​GCA​GGG​AGG​AAA​AG-3′, *Iba1*/*Aif1* reverse: 5′-TGG​GAT​CAT​CGA​GGA​ATT​G-3′.

*Cd68 forward*: 5′-GAC​ACT​TCG​GGC​CAT​GTT-3′, *Cd68* reverse: 5′-GAG​GAG​GAC​CAG​GCC​AAT-3.’

*Gfap* forward: 5′-GGA​GAG​GGA​CAA​CTT​TGC​AC-3′, *Gfap* reverse: 5′-AGC​CTC​AGG​TTG​GTT​TCA​TC-3′.

*C9orf72 G*_*4*_ *C*_*2*_ forward: 5′-TAGTACTCGCTGAGGGTGAAC-3′, *C9orf72 G*_*4*_ *C*_*2*_ reverse: 5′-CTA​CAG​GCT​GCG​GTT​GTT​TC-3′.

*Tardbp* forward: 5′-GGG​GCA​ATC​TGG​TAT​ATG​TTG-3′, *Tardbp* reverse: 5′-TGG​ACT​GCT​CTT​TTC​ACT​TTC​A-3’.

### Protein extraction and immunoblotting

To prepare protein lysates from mouse brains, Triton X-100 and Sodium dodecyl sulfate (SDS) were added to brain homogenates at final concentrations of 1% and 2%, respectively. Homogenates were sonicated on ice and centrifuged at ×16,000 g for 20 min. The supernatants were collected, and protein concentrations were determined by a bicinchoninic acid (BCA) assay (ThermoFisher Scientific). For human post-mortem frontal cortex samples, 50 mg of tissue was homogenized in Radioimmunoprecipitation buffer (RIPA, 25 mM Tris-HCl pH 7.6, 150 mM NaCl, 1% sodium deoxycholate, 1% Nonidet P-40, 0.1% sodium dodecyl sulfate, protease, and phosphatase inhibitors). Tissue homogenates were sonicated on ice and centrifuged at ×100,000 g for 30 min at 4°C. The supernatant was collected and is referred to as the RIPA-soluble fraction, with protein concentration determined by BCA assay. The RIPA-insoluble pellet was extracted using 7 M urea buffer, sonicated, and then centrifuged for 30 min at ×100, 000 g at room temperature. Protein concentrations of the urea-soluble fractions were determined by Bradford assay.

Protein lysates were diluted in Tris-Glycine SDS sample buffer (ThermoFisher Scientific) and 5% beta-mercaptoethanol (Sigma Aldrich). Protein samples were heat denatured for 5 min at 95°C and subjected to sodium dodecyl sulfate–polyacrylamide gel electrophoresis (SDS-PAGE) on 4%–20% Tris-Glycine gels (Life Technologies). Protein was subsequently transferred to Polyvinylidene (PVDF) membrane (Millipore). Membranes were first blocked with 5% non-fat dry milk in Tris-buffered saline with Triton (TBS-T, 100 mM Tris-HCl pH 7.5, 140 mM NaCl, 0.1% Triton X-100) and incubated overnight at 4°C with mouse anti-STMN2 (1:1000 MAB6930, R&D Systems), rabbit anti-TDP-43 (1:5,000 12892-AP ProteinTech) and mouse monoclonal anti-glyceraldehyde-3-phosphate dehydrogenase (GAPDH) antibody (1:30,000, H86504M, Meridian Life Sciences), diluted in TBS-T plus 5% nonfat dry milk. Membranes were then incubated with horseradish peroxidase (HRP)-conjugated secondary antibodies (1:5,000; Jackson ImmunoResearch) and signal was detected by Enhanced chemiluminescence (ECL, PerkinElmer). Quantitative densitometry was performed using ImageJ, and Stmn2 levels were normalized to Gapdh expression.

### Immunohistochemistry

Paraffin-embedded brains were deparaffinized in xylene and rehydrated through a series of ethanol solutions. Antigen retrieval was performed by steaming in sodium citrate buffer (10 mM sodium citrate, 0.05% Tween-20, pH 6), distilled water, or pH 9 Tris-EDTA for 30 min. Tissues were immunostained with polyclonal antibodies against poly(GP) [1:10,000, Rb5823 (Gendron et al., 2013)] and Gfap (1:2,500, BioGenex). For phosphorylated Tdp-43 (pTdp-43) staining, tissue sections were incubated with Dual Endogenous Enzyme Block (DAKO) and blocked with 2% normal goat serum. Slides were incubated with our novel MA1290/1291AP in-house antibody (1:100), an anti-rabbit antibody for both mouse and human TDP-43 phosphorylated at serine 409/410, overnight at 4°C. The recognized antigen is amino acid residues 404 to 414 of full-length TDP-43 with phosphorylation of S409/410 (CSMDSK[pS][pS]GWGM), and equivalent to our previously described Rb3655 antibody (Todd et al., 2020; Wu et al., 2021). The following day, slides were washed and incubated with biotinylated goat anti-rabbit secondary (1:200, Vectastain^®^ ABC-HRP Kit, Peroxidase (Rabbit IgG) PK-4001) for 2 h at room temperature. Slides were incubated with avidin-biotin complex solution for 30 min. Slides were then incubated with 3,3′-diaminobenzidine (DAB, Acros Organics) activated with hydrogen peroxide, and the reaction was stopped by rinsing slides in distilled water. Sections were counterstained with hematoxylin, dehydrated through a series of ethanol and xylene washes, and coverslipped with Cytoseal mounting media (Thermo Fisher Scientific).

### Quantification of neuropathology

The burden of GP inclusions and Gfap immunoreactivity was quantified using digital pathology technology by Leica Biosystems. We used a ScanScope^®^ AT2 (Leica Biosystems) to obtain high-resolution digitized images of immunostained slides. ImageScope^®^ software (v12.1; Leica Biosystems) was used to annotate the cortex on mid-sagittal serial sections stained for GP of Gfap for each mouse. A custom-designed Positive Pixel Count algorithm was used to quantify either poly-GP burden or Gfap immunoreactivity in the cortex. pTdp-43 inclusions in the cortex of each mouse sample were counted manually in a blinded fashion, with the number of pTdp-43 inclusions per mm^2^ determined for each slide.

### Immunoassays for dipeptide repeat proteins and phosphorylated TDP-43

Previously characterized Meso Scale Discovery (MSD) sandwich immunoassays were used to quantify poly (GP) and poly (GA) (Gendron et al., 2015) from mouse brains. The abundance of pTDP-43 in the urea-soluble fraction from the frontal cortex of human cases and controls had been measured in a previous study by sandwich Meso Scale Discovery (MSD) immunoassay (Prudencio et al., 2020). Lysates were diluted in Tris buffered saline (TBS) and an equal amount of protein was loaded in duplicate wells. Response values corresponding to the intensity of emitted light upon electrochemical stimulation of the assay plate using the MSD QUICKPLEX SQ120 were acquired.

### Statistics

GraphPad Prism 9 (GraphPad software) was used for all statistical analyses. The type of statistical analysis used, the number of subjects, and the *p* values used are indicated in the figure and/or legend. To compare STMN2 protein abundance in FTD and controls, the data was analyzed with a single-variable (unadjusted) and a multivariable linear regression model (adjusted), considering age at death and sex. *p*-values less than 0.0125 were considered statistically significant, after comparing the four groups, all FTD combined and each genotype separately. To compare pTDP-43 abundance between FTD groups, that data was analyzed using both single-variable (unadjusted) and multivariable linear regression (adjusted) models, factoring in age at death and sex. *p*-values less than 0.025 were considered statistically significant after multiple comparisons. Associations between STMN2 protein and TDP-43 or pTDP-43 protein levels in FTD cases were determined using a single-variable and multivariable linear regression model, the latter was adjusted for age at death and sex. *p*-values less than 0.025 were considered statistically significant after multiple comparisons. Due to their skewed distribution, STMN2, TDP-43 and pTDP-43 protein levels were plotted on a base 2 logarithmic scale.

## Results

### STMN2 protein is reduced in the frontal cortex of familial FTD cases

**FIGURE 1:**
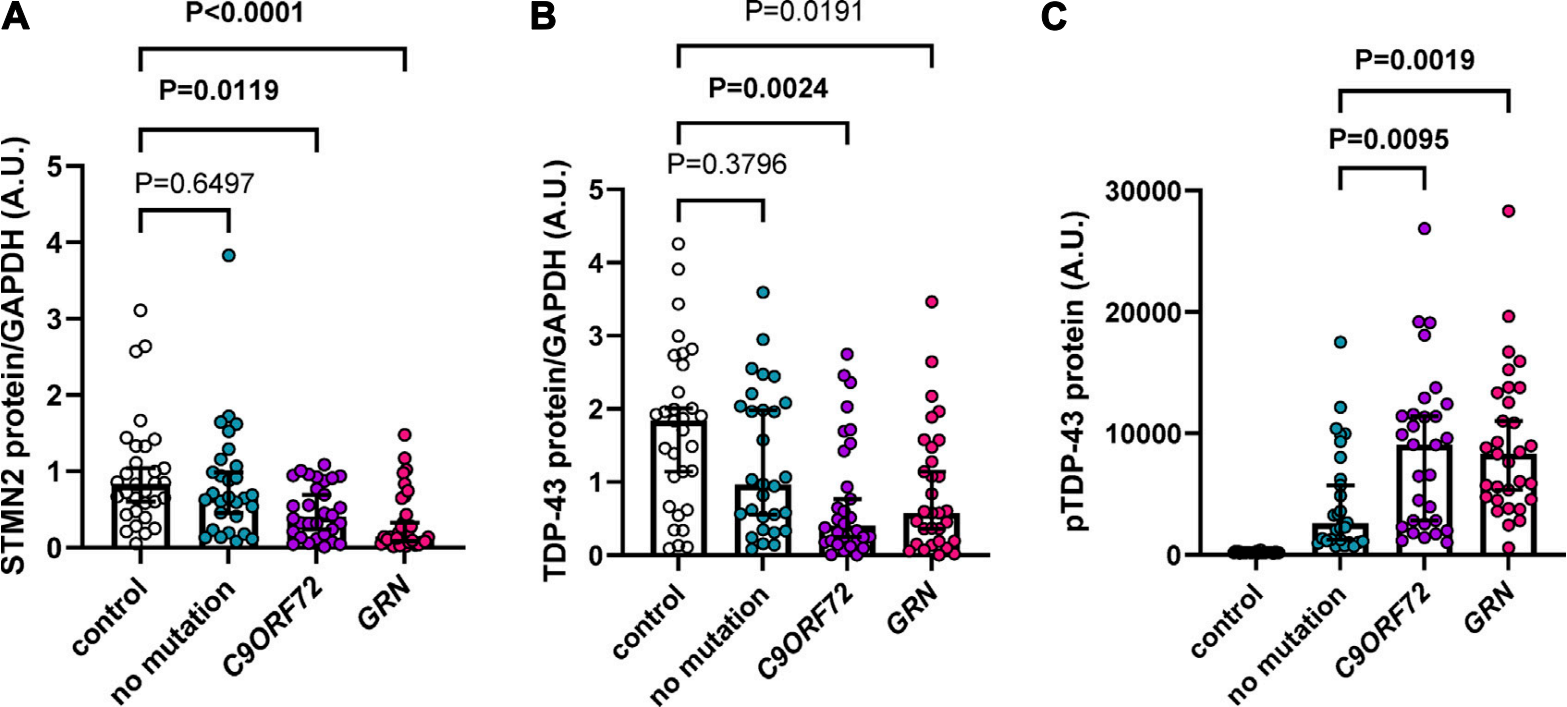
Soluble STMN2 and TDP-43 protein are reduced in the frontal cortex of familial FTLD-TDP cases. **(A,B)** Soluble STMN2 **(A)**, TDP-43 **(B)** protein levels were measured by Western blot in the frontal cortex of FTLD-TDP cases (*n* = 90) and non-neurological disease controls (*n* = 31). Following quantification of STMN2 **(A)** or TDP-43 **(B)** immunoreactivity, levels were normalized to GAPDH to control for protein loading. **(C)** pTDP-43 protein levels, as measured by immunoassay, are significantly elevated in cases with a *C9ORF72* repeat expansion or *GRN* mutations compared to those without mutations. Horizontal bars represent median ± 95% confidence intervals. *p*-values from the linear regression model adjusting for age and sex are shown. Values for both unadjusted and adjusted linear regression models are reported in Supplementary Tables S2, S4.

Previous studies demonstrated a reduction of *STMN2* RNA in FTD and ALS cases (Klim et al., 2019; Melamed et al., 2019; Prudencio et al., 2020) and a decrease in STMN2 immunoreactivity in motor neurons from ALS cases compared to controls (Klim et al., 2019). However, STMN2 protein levels in FTD cases have yet to be evaluated. To do so, we assembled an FTD cohort with a neuropathological diagnosis of frontotemporal lobar degeneration with TDP-43 pathology (FTLD-TDP), with an approximately equal number of cases having an expanded G_4_C_2_ repeat in the *C9ORF72* gene, *GRN* mutations or no known FTD-causative mutation (Supplementary Table S1). We then measured detergent-soluble STMN2 protein in the frontal cortex by Western blot, which revealed a significant or nominally significant reduction of STMN2 protein in all FTLD-TDP cases compared to controls in unadjusted analysis and adjusted analysis when controlling for age and sex, respectively (Figure 1A; Supplementary Table S2). Lower levels of STMN2 protein in the FTLD-TDP group were largely driven by carriers of a *C9ORF72* expanded repeat and *GRN* mutations, as individuals without a mutation had similar levels of STMN2 protein compared to controls. We speculated that cases harboring mutations may have greater reductions in STMN2 protein due to lower levels of soluble TDP-43 protein. To that end, we measured soluble TDP-43 levels in our cohort of controls and FTLD-TDP cases. Compared to controls, we observed a significant or nominally significant reduction in TDP-43 levels in unadjusted and analysis adjusted for age and sex, respectively (Figure 1B; Supplementary Table S2). Like STMN2 levels, the reduction in soluble TDP-43 protein was more pronounced in mutation carriers. Additionally, lower levels of TDP-43 protein were significantly associated with reduced levels of soluble STMN2 in FTLD-TDP cases in both unadjusted and adjusted analyses controlled for age and sex (Supplementary Table S3). We further suspected that levels of pTDP-43 pathology may also be greater in mutation carriers. To evaluate this possibility, we used pTDP-43 protein levels we had measured in these same cases from a previous study (Prudencio et al., 2020) and compared the pTDP-43 burden in sporadic and familial FTLD-TDP cases. Indeed, mutation carriers had significantly elevated levels of pTDP-43 than those without, in both unadjusted and adjusted analysis (Figure 1C; Supplementary Table S4), in line with our previous findings (Prudencio et al., 2017). Higher levels of pTDP-43 were found to have a significant or nominally significant association with lower levels of STMN2 protein in FTLD-TDP cases (Supplementary Table S3). Taken together, we provide evidence of decreased STMN2 and TDP-43 abundance in FTLD-TDP cases harboring *C9ORF72* and *GRN* mutations. These findings suggest that loss of STMN2 protein is a feature of familial FTD and may contribute to pathogenesis.

### Creation of a Stmn2 loss of function model using CRISPRi

**FIGURE 2:**
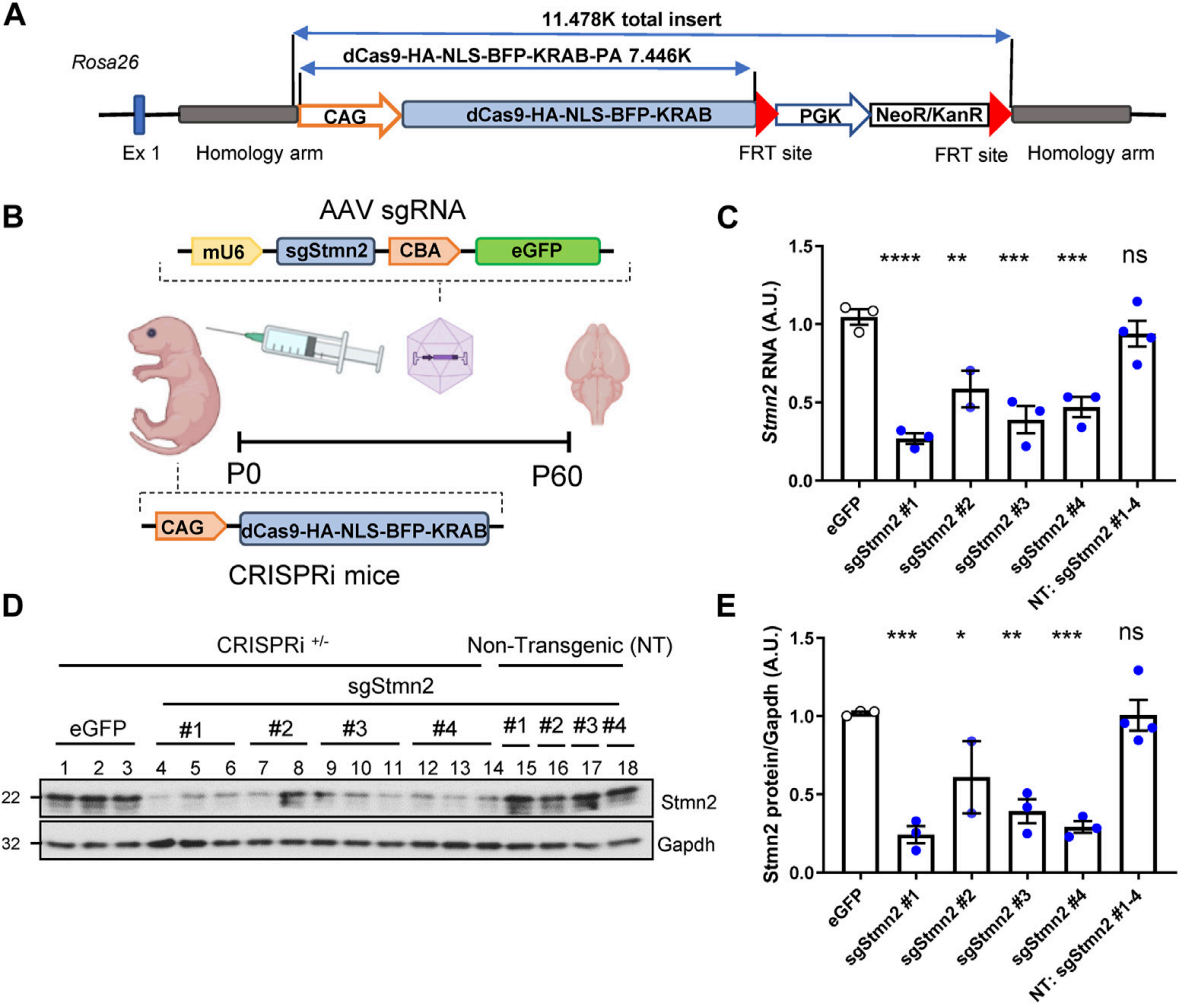
Generation of Stmn2 knockdown mouse model using CRISPRi. **(A)** Schematic of CRISPRi machinery used to create CRISPRi knockin mice. **(B)** Schematic of CRISPRi mice, AAV sgRNA construct, and delivery of virus by ICV injection to create an *in vivo* model of Stmn2 loss of function. **(C–E)** Stmn2 RNA and protein levels were assessed in the brain tissue of 2-month-old CRISPRi mice and/or non-transgenic littermate controls injected with AAV encoding eGFP, or four guides targeting Stmn2 (sgStmn2#1-4). **(C)** *Stmn2* RNA levels, normalized to endogenous controls *Rplp0* and *Gapdh,* as measured by qRT-PCR, are significantly reduced in CRISPRi mice injected with AAV sgStmn2. Immunoblot **(D)** and quantification of Stmn2 protein normalized to Gapdh levels **(E)** reveal a reduction of Stmn2 protein in CRISPRi mice injected with sgStmn2. Data presented as mean ± SEM. *p < 0.05, **p < 0.01, ***p < 0.001, ****p < 0.0001, non-significant (ns), as analyzed using one-way ANOVA, followed by Dunnett’s *post hoc* analysis, *n* = 2–4 animals per group **(C,E)**. Schematic **(B)** created with BioRender.com.

To model Stmn2 loss of function *in vivo* and assess its relevance in FTD, we first generated a novel knockin CRISPRi mouse model using a system that previously demonstrated effective silencing of target genes *in vitro* (Gilbert et al., 2014). The CRISPRi machinery was integrated into the *Rosa26* locus and expresses a catalytically dead form of Cas9 (dCas9) fused to a transcriptional repressor domain, KRAB (Figure 2A). AAV was used to deliver sgRNA to direct CRISPRi machinery to the *Stmn2* transcription start site (Figure 2B). We tested four sgRNAs targeted against Stmn2 from a CRISPRi library of the whole mouse genome (Horlbeck et al., 2016). To deliver guides to the mouse brain, ICV injections of AAV sgStmn2 (#1–4) were performed at postnatal day zero, and mice were harvested at 2 months of age (Figure 2B). Notably, all four guides were able to significantly reduce *Stmn2* RNA (Figure 2C) and protein (Figures 2D, E) compared to CRISPRi mice injected with enhanced Green Fluorescent Protein (eGFP) or non-transgenic mice (NT, lacking the CRISPRi machinery). While Stmn2 protein knockdown ranged from 40% to 75%, mice injected with sgStmn2#1 displayed the largest reduction in Stmn2 protein (Figures 2D, E). We therefore selected sgStmn2#1 for further experiments and will refer to it as sgStmn2 hereafter. We were also able to modulate the levels of Stmn2 knockdown by titrating the amount of virus delivered, with the highest levels of virus showing the largest decrease in both Stmn2 RNA (Supplementary Figure S1A) and protein levels (Supplementary Figures S1B, C). Taken together, we were able to achieve robust knockdown of Stmn2 in the brain of CRISPRi mice.

### Establishing a dual CRISPRi Stmn2 loss of function and *C9ORF72* expanded repeat model

Our group has previously shown that in comparison to mice expressing a non-pathological number of 2 G_4_C_2_ repeats (2R), mice expressing 149 G_4_C_2_ repeats (149R) recapitulate C9FTD relevant disease features including pTdp-43 inclusions, accumulation of RNA foci and DPR proteins from both sense and antisense transcripts, neurodegeneration and behavioral deficits (Chew et al., 2015; Chew et al., 2019). To understand if Stmn2 deficiency would impact *C9ORF72-*associated pathology we used AAV to simultaneously express either sgStmn2 or a non-targeting guide RNA (sgCtrl) along with either 2R or 149R.

**FIGURE 3:**
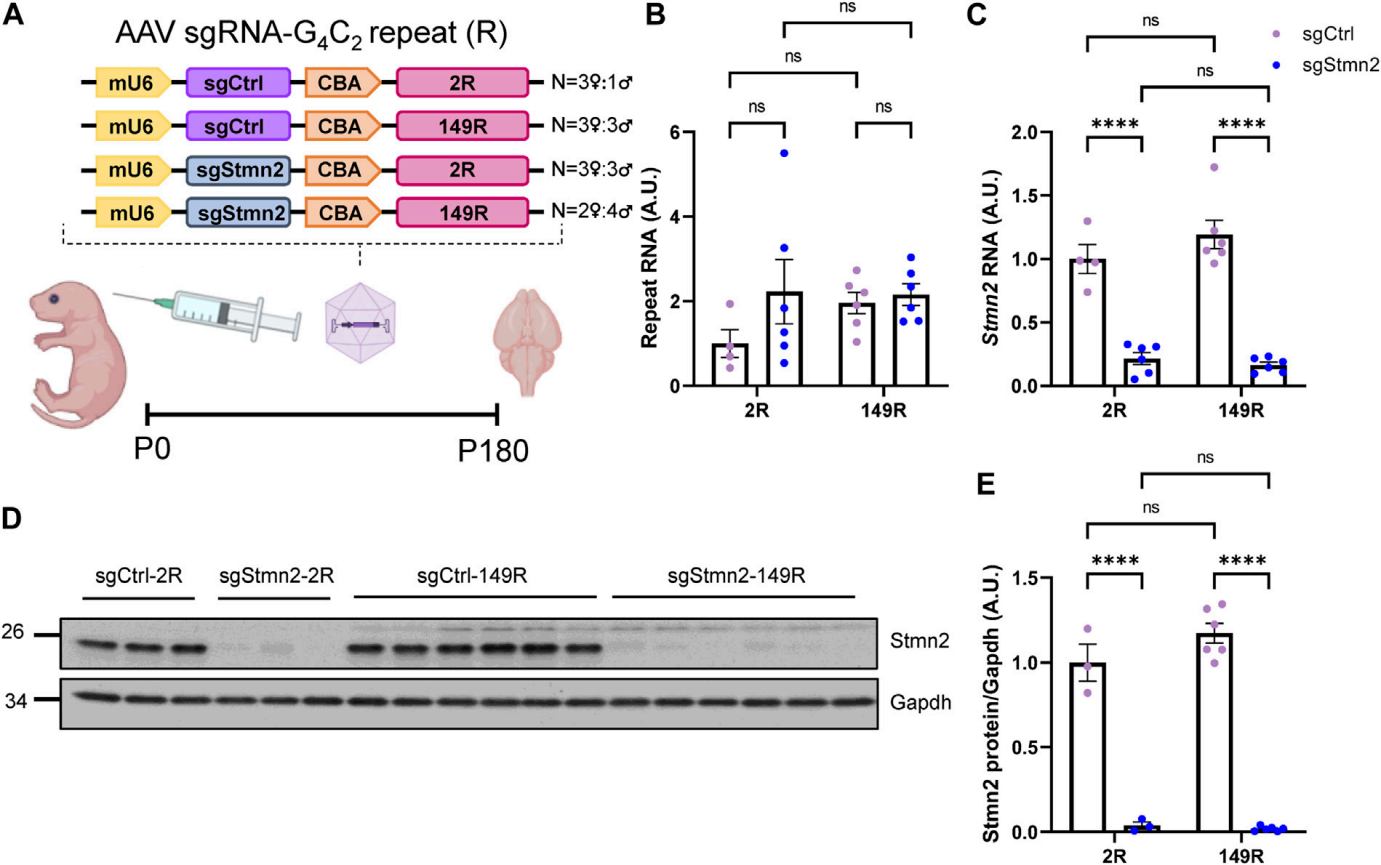
Generation of dual *C9ORF72* expanded repeat and Stmn2 loss of function model in CRISPRi mice. **(A)** Schematic of AAV sgRNA-G_4_C_2_ repeat construct and delivery of virus to create a dual model. **(B–E)** The levels of the *C9ORF72* G_4_C_2_ repeat **(B)**, *Stmn2* RNA **(C)**, and Stmn2 protein **(D,E)** were measured in brain tissue of 6-month-old CRISPRi mice injected with AAV encoding sgCtrl or sgStmn2, with either 2 (2R) or 149 G_4_C_2_ repeats (149R). **(B)** Levels of G_4_C_2_ repeat RNA are similar regardless of sgRNA or repeat expression. **(C)** *Stmn2* RNA levels, normalized to endogenous controls *Rplp0* and *Gapdh* as measured by qRT-PCR, are significantly reduced in CRISPRi mice receiving sgStmn2 compared to sgCtrl, regardless of repeat number. Immunoblot **(D)** and quantification of Stmn2 protein normalized to Gapdh levels **(E)** reveal a significant reduction of Stmn2 protein in CRISPRi mice injected with sgStmn2 compared to sgCtrl, regardless of repeat number. Data presented as mean ± SEM. ****p < 0.0001, non-significant (ns), as analyzed using two-way ANOVA, followed by Tukey’s *post hoc* analysis, *n* = 3–6 animals per group **(B–E)**. Schematic **(A)** created with BioRender.com.

Each AAV (sgCtrl-2R, sgCtrl-149R, sgStmn2-2R, and sgStmn2-149R) was introduced to the mouse CNS via ICV injection at postnatal day 0 (Figure 3A). Animals were harvested at 6 months of age, a time point when we previously observed robust pathology and gliosis in the 149R-AAV model (Chew et al., 2019). We first confirmed the expression of the G_4_C_2_ repeat transcript, with similar levels observed between all four groups (Figure 3B). Moreover, as anticipated, *Stmn2* RNA levels were significantly reduced in mice injected with sgStmn2 compared to mice injected with the non-targeting sgCtrl (Figure 3C). Stmn2 protein levels were also significantly reduced in sgStmn2-2R and sgStmn2-149R mice (Figures 3D, E). Of note, we did not observe any differences in Stmn2 knockdown efficiency as a function of the G_4_C_2_ repeat number. We also measured transcripts of other stathmin family genes to both verify the specificity of the guide RNA for Stmn2 and exclude any possible compensatory upregulation mechanisms (Gagliardi et al., 2022). Analysis of *Stmn1*, *3*, or *4* transcript levels revealed no significant differences among any of the four groups (Supplementary Figures S2A–C).

### Dual CRISPRi Stmn2 loss of function and *C9ORF72* expanded repeat model recapitulates C9-associated pathology

**FIGURE 4:**
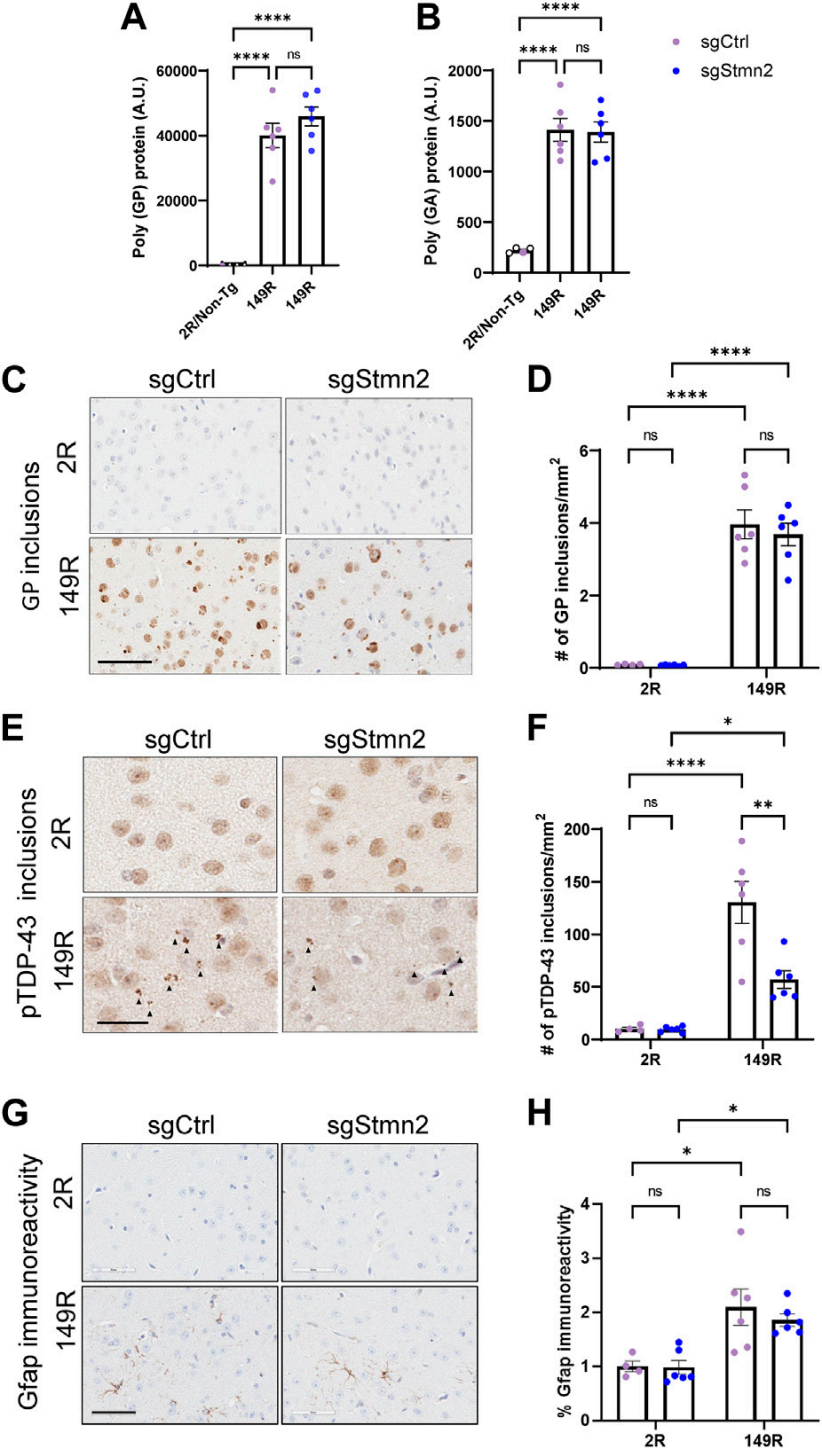
Dual *C9ORF72* expanded repeat and Stmn2 loss of function model recapitulates C9-associated pathology. **(A–H)** DPR accumulation, pTdp-43 pathology, and gliosis were analyzed in the brains of 6-month-old mice. **(A,B)** Immunoassay measuring soluble poly-GP **(A)** and poly-GA **(B)** show a significant accumulation of DPRs in sgCtrl-149R and sgStmn2-149R mice. **(C)** Representative images of immunohistochemical analysis of poly(GP) with quantitative analysis of the number of GP-positive inclusions in the cortex **(D)**. **(E)** Representative images of pTdp-43 immunoreactivity and quantitative analysis of the number of pTdp-43 inclusions observed in the cortex **(F)**. **(G)** Representative images of immunohistochemical analysis of Gfap levels with quantitative analysis of percent Gfap immunoreactivity **(H)**. Data presented as mean ± SEM. *p < 0.05, **p < 0.01, ****p < 0.0001, non-significant (ns), as analyzed using two-way ANOVA, followed by Tukey’s *post hoc* analysis, *n* = 4–6 animals per group **(A–H)**. Scale bar is 100 uM **(C)** and 60 uM **(E,G)**. Solid arrows indicate pTdp-43 inclusions **(E)**.

To validate whether our CRISPRi dual model replicated *C9ORF72* expanded repeat-associated pathology, we assessed DPR protein expression and pTdp-43 accumulation. First, we measured the expression of DPR proteins in the cortex using established immunoassays (Chew et al., 2019). As anticipated, we detected robust expression of poly(GP) (Figure 4A) and poly(GA) (Figure 4B) in sgCtrl-149R and sgStmn2-149R mice compared to animals not expressing the expanded repeat. Reduction of Stmn2 protein levels did not impact either poly(GA) or poly (GP) protein levels. Immunohistochemical analysis using a poly(GP)-specific antibody revealed both diffuse nuclear staining in the cortex as well as cytoplasmic inclusions that were restricted to animals expressing the expanded repeat (149R) (Figures 4C, D). The number of GP inclusions was similar between sgCtrl-149R and sgStmn2-149R animals consistent with our immunoassay results.

We next evaluated pTdp-43 inclusions and observed small cytoplasmic inclusions in the cortex of sgCtrl-149R mice that were absent in sgCtrl-2R mice (Figures 4E, F). Unexpectedly, we found fewer pTdp-43 inclusions in sgStmn2-149R mice compared to sgCtrl-149R mice (Figure 4F) in the absence of changes in *Tardbp* RNA levels (Supplementary Figure S3A).

We also assessed gliosis in the cortex and observed a significant increase in Gfap immunoreactivity, a marker of activated astrocytes, (Figures 4G, H), in mice expressing the *C9ORF72* expanded repeat, in line with our previous mouse model (Chew et al., 2019). Levels of the *Gfap* transcript were also significantly elevated in the 149R groups (Supplementary Figure S4A). Additionally, RNA levels of markers of activated microglia, (*Iba1/Aif1*, Supplementary Figure S4B), and phagocytic microglia (*Cd68*, Supplementary Figure S4C) were significantly increased in groups expressing the expanded repeat, suggestive of microgliosis, although this was not confirmed at the protein level. Reduction of Stmn2 protein did not consistently induce or exacerbate gliosis. However, we did observe a significant increase in *Cd68* transcript levels in the sgStmn2-2R group compared to sgCtrl-2R (Supplementary Figure S4C). A significant increase of *Gfap* RNA levels was observed in Stmn2 depleted mice (sgStmn2-149R) compared to controls (sgCtrl-149R) in animals expressing the expanded repeat (Supplementary Figure S4A), although this was not preserved at the protein level (Figure 4H). Together, these data provide proof-of-concept that we were able to successfully deliver guide RNA to knock down a target of interest in CRISPRi mice in a disease model (in this case mice expressing the expanded *C9ORF72* repeat). Overall, loss of Stmn2 in the presence of the *C9ORF72* expanded repeat does not exacerbate C9-mediated pathology at 6 months of age in the current model.

## Discussion

Previous studies had suggested STMN2 is integral for axon growth, maintenance, and stability (Shin et al., 2012; Shin et al., 2014). Loss of Stmn2 expression in fly and mouse models have identified a role for Stmn2 in motor coordination, innervation of the NMJ, and neuronal survival of dopaminergic neurons in the substantia nigra (Graf et al., 2011; Duncan et al., 2013; Wang et al., 2019; Guerra San Juan et al., 2022; Krus et al., 2022; Lopez-Erauskin et al., 2022). The discovery that TDP-43 regulates STMN2 processing and that *STMN2* RNA levels are reduced in ALS/FTD cases have fueled speculation of its involvement in the pathogenesis of these diseases. Recent evidence from three Stmn2 loss of function mouse models strongly support that the loss of STMN2 contributes to ALS (Guerra San Juan et al., 2022; Krus et al., 2022; Lopez-Erauskin et al., 2022). However, the role of STMN2 in the central nervous system beyond the spinal cord and its relevance to FTD remain unresolved. We find that soluble STMN2 and TDP-43 protein levels are significantly reduced in the frontal cortex of FTD cases with *GRN* mutations and a *C9ORF72* repeat expansion, these findings support the notion that STMN2 deficiency is a feature of familial FTD. However, the specific contribution of STMN2 loss in the pathogenesis of FTD has yet to be delineated. Regardless, these data support the development of therapies aimed at restoring STMN2 levels in FTD with TDP-43 pathology. Indeed, antisense oligonucleotides (ASO) are attractive therapeutics for the treatment of neurodegenerative diseases following the promising clinical trial results and Federal Drug Administration approval of nusinersen for the treatment of Spinal Muscular Atrophy (Schoch and Miller, 2017; Mercuri et al., 2018). ASOs to correct *STMN2* splicing and augment protein levels were shown effective in TDP-43 depleted iPSC-derived motor neurons, TDP-43 depleted primary cortical neurons from mice with a humanized *STMN2* gene and in mice with constitutive misprocessing of the human *STMN2* gene (Baughn et al., 2023). Given these compelling pre-clinical results, phase 1 trials using ASOs to restore *STMN2* splicing levels in ALS patients are currently underway (Corporation, 2022). ASOs to restore STMN2 levels in FTD are also in the preclinical phase.

To provide a tool to rapidly interrogate the contribution of loss of function of a target of interest we generated a novel CRISPRi knockin mouse model and used knockdown of Stmn2 as a test-case. We achieved a robust knockdown of Stmn2 RNA and protein, which can be titrated either by the selection of guide RNA or the amount of virus. As multiple human diseases are caused by haploinsufficiency (Dang et al., 2008), including FTD with *GRN* mutations (Rhinn et al., 2022), the ability to fine-tune the level of knockdown by either guide design or the amount of AAV delivered will be of great value. Further, AAV delivery of guide RNA offers long-term gene silencing compared to ASOs (Murlidharan et al., 2014). In addition, knockdown is achieved postnatally, circumventing embryonic or perinatal lethality if the target of interest is required during embryogenesis or development. Indeed, Stmn2 likely plays an unresolved role in early development, as two groups reported perinatal lethality in homozygous Stmn2 knockouts (Krus et al., 2022; Lopez-Erauskin et al., 2022) or mice with constitutive human STMN2 processing defects (Baughn et al., 2023).

Genetic screens in fly and yeast have identified the nuclear pore complex and nuclear export/import factors as possible modifiers of *C9ORF72*-mediated toxicity (Freibaum et al., 2015; Jovicic et al., 2015; Zhang et al., 2015; Boeynaems et al., 2016), however these candidates have yet to be evaluated *in vivo* or in C9FTD/ALS mouse models. Likewise, CRISPR/Cas9 screens examining modifiers of DPR toxicity (Kramer et al., 2018) or RAN-translation (Cheng et al., 2019) have also produced promising candidate genes requiring validation in preclinical animal models. Evaluation of promising candidates using the CRISPRi mouse could serve as a natural extension of CRISPR screens in human cell lines. Taken together, the CRISPRi model offers high flexibility in target selection, can be rapidly generated, and is more economical compared to conventional knockouts.

We also created a dual model where Stmn2 protein deficiency was combined with the expression of a *C9ORF72* expanded repeat. DPR accumulation was similar between sgCtrl-149R and sgStmn2-149R animals as evidenced by immunoassay and immunohistochemical analysis. Unexpectedly, we did observe a significant decrease in the number of pTdp-43 inclusions in the cortex of sgStmn2-149R mice. Modulating Stmn2 expression has been reported to alter levels of other neurogenerative disease proteins, although the mechanisms are not defined. Knockdown of Stmn2 in the midbrain has been shown to lead to increased levels of phosphorylated alpha-synuclein (Wang et al., 2019). In addition, overexpression of STMN2 in the hippocampus of an Alzheimer’s disease mouse model resulted in decreased amyloid β (Aβ) staining and plaque burden, presumably through STMN2-dependent regulation of the intracellular trafficking and processing of amyloid precursor protein (APP) (Wang et al., 2013). TDP-43 regulates STMN2 expression, but in turn, it is possible that Stmn2 affects pTdp-43 levels either directly or indirectly. Alternatively, the accumulation of pTdp-43, which correlates with neurodegeneration in C9FTD (Mackenzie et al., 2013), and loss of Stmn2 may synergize and lead to neuronal loss. At present, we did not test this speculation directly, as reports on the requirement for Stmn2 for neuronal survival have been mixed. Depletion of Stmn2 in the substantia nigra led to a marked decrease of dopaminergic hydroxylase positive (TH+) neurons, but not overall neuron counts (Wang et al., 2019). Despite motor impairments and denervation at the NMJ, Stmn2 knockouts did not display overt neurodegeneration of motor neurons in the spinal cord (Guerra San Juan et al., 2022; Krus et al., 2022; Lopez-Erauskin et al., 2022). These findings may indicate that STMN2 expression may be required for specific neuronal subpopulations. Further study is required to clarify the link between Stmn2 and levels of TDP-43 and other disease-associated proteins. Suppressing expression of the splicing factor SYF2 in a mouse model overexpressing human TDP-43 can rescue motor deficits and TDP-43 pathology (Linares et al., 2023). Depletion of SYF2 in the context of the CRISPRi *C9ORF72* model could extend these observations to FTD. Beyond *C9ORF72*-mediated FTD, we anticipate this model may be useful to interrogate newly identified risk factors and disease modifiers in other neurodegenerative diseases. Indeed, our group has reported Spinocerebellar Ataxia (SCA3) (Jansen-West et al., 2022) and Tau (Cook et al., 2015) models which could be combined with knockdown of disease modifiers in our CRISPRi mouse. This and other CRISPRi mouse models could be a first step toward the development of more complex gene silencing models, such as cell-type specific expression of the dCas9 machinery to rapidly determine cell-autonomous and non-cell autonomous gene function, or inducible expression of repression machinery to compare gene silencing early and late in disease course.

Despite the advantages of the CRISPRi model our study did have limitations. We were able to achieve a robust knockdown of Stmn2 RNA and protein in the dual CRISPRi model, but we did not eliminate expression. Therefore, it is possible that the remaining Stmn2 protein levels were sufficient to carry out its function in the cortex. Stmn2^+/−^ heterozygous mice also exhibit deficits in motor behavior and neuromuscular innervation that only became evident with advanced age (12 months compared to 3 months in homozygous mice) (Krus et al., 2022). Denervation in the heterozygote was also less severe than the Stmn2 knockout (Krus et al., 2022), suggesting that even low levels of Stmn2 can maintain synaptic integrity. We assessed pathology in the cortex at 6 months of age, which may not be sufficient if the effects of Stmn2 depletion are age dependent. We did not characterize FTD-related behavioral deficits nor neurodegeneration in this proof-of-concept study. These would be evident next steps, which may shed light on the role of Stmn2 in brain regions beyond the spinal cord.

Our findings of reduced STMN2 protein levels in *C9ORF72* and *GRN-*linked FTD support efforts focused on determining the contribution of STMN2 deficiency in FTD pathogenesis. Additionally, our results provide a rationale for the development and use of ASOs to correct *STMN2* splicing for individuals with FTD, particularly those harboring genetic mutations. Further, we provide proof-of-concept that CRISPRi-mediated knockdown in a disease model is feasible, demonstrating the ability to suppress Stmn2 expression alone and in combination with the *C9ORF72* expanded repeat. Further, our dual model replicates disease features previously reported in a *C9ORF72* mouse. We propose that CRISPRi mice provide a versatile and rapid method to silence the expression of novel targets of interest and will be useful to interrogate gene function alone or in the context of other disease models *in vivo*. We anticipate this model will accelerate the translation of newly identified targets to preclinical disease models and ultimately to the clinic.

## Data Availability

The raw data supporting the conclusion of this article will be made available by the authors, without undue reservation.

## Supplementary material

SUPPLEMENTARY FIGURE S1 Knockdown of Stmn2 can be modulated in CRISPRi mice. **(A)** Stmn2 RNA levels were measured by qRT-PCR in 2-month-old CRISPRi mice injected with AAV encoding sgCtrl or sgStmn2 at various titers. **(B,C)** Stmn2 protein levels were examined by immunoblot **(B)**, followed by quantification and normalization to Gapdh to control for protein loading **(C)**. Data presented as mean ± SEM. **p* < 0.05, ***p* < 0.01, ****p* < 0.001, *****p* < 0.0001, non-significant (ns), with analysis performed by one-way ANOVA followed by Tukey’s post hoc analysis, *n* = 2–3 animals per group **(A,C)**.

SUPPLEMENTARY FIGURE S2 Knockdown of Stmn2 does not lead to upregulation of Stathmin family genes. **(A-C)** The levels of *Stmn1* **(A)**, *Stmn3* **(B)**, and *Stmn4* RNA **(C)** were measured by qRT-PCR and normalized to endogenous controls *Rplp0* and *Gapdh*, in brain tissue of 6-month old CRISPRi mice injected with AAV encoding sgCtrl or sgStmn2 with either 2 or 149 G_4_C_2_ repeats. Data presented as mean ± SEM. non-significant (ns), as analyzed using two-way ANOVA, followed by Tukey’s *post hoc* analysis, *n* = 4–6 animals per group.

SUPPLEMENTARY FIGURE S3 Knockdown of Stmn2 does not impact *Tardbp* levels. **(A)** The levels of *Tardbp* were measured by qRT-PCR and normalized to endogenous controls *Rplp0* and *Gapdh* in brain tissue of 6-month-old CRISPRi mice injected with AAV encoding sgCtrl or sgStmn2 with either 2 or 149 G_4_C_2_ repeats. Data presented as mean ± SEM. non-significant (ns), as analyzed using two-way ANOVA, followed by Tukey’s *post hoc* analysis, *n* = 4–6 animals per group.

SUPPLEMENTARY FIGURE S4 Transcript levels of glial markers are increased in CRISPRi mice expressing a *C9ORF72*-expanded repeat. RNA levels of glial markers were assessed by qRT PCR and normalized to endogenous controls *Rplp0* and *Gapdh*, revealing a significant increase in *GFAP* levels **(A)**, *Iba1/Aif1* levels **(B)**, and *Cd68* levels **(C)** in mice expressing the expanded repeat. Data presented as mean ± SEM. **p* < 0.05, ****p* < 0.001, *****p* < 0.0001, non-significant (ns), as analyzed using two-way ANOVA, followed by Tukey’s *post hoc* analysis, *n* = 4–6 animals per group.

SUPPLEMENTARY TABLE S1 Characteristics of controls and FTLD-TDP cases.

SUPPLEMENTARY TABLE S2 Comparison of STMN2 and TDP-43 protein levels in the frontal cortex of controls and FTLD-TDP cases.

SUPPLEMENTARY TABLE S3 Association of STMN2 protein with TDP-43 and pTDP-43 protein levels in FTLD-TDP cases.

SUPPLEMENTARY TABLE S4 Comparison of pTDP-43 protein levels between FTLD-TDP cases.
