# Supplementary figures and images for: CRISPR interference to evaluate modifiers of C9ORF72-mediated toxicity in FTD
Source: Front Cell Dev Biol. 2023 Aug 7;11:1251551. doi: 10.3389/fcell.2023.1251551 (PMC10443592; doi:10.3389/fcell.2023.1251551)

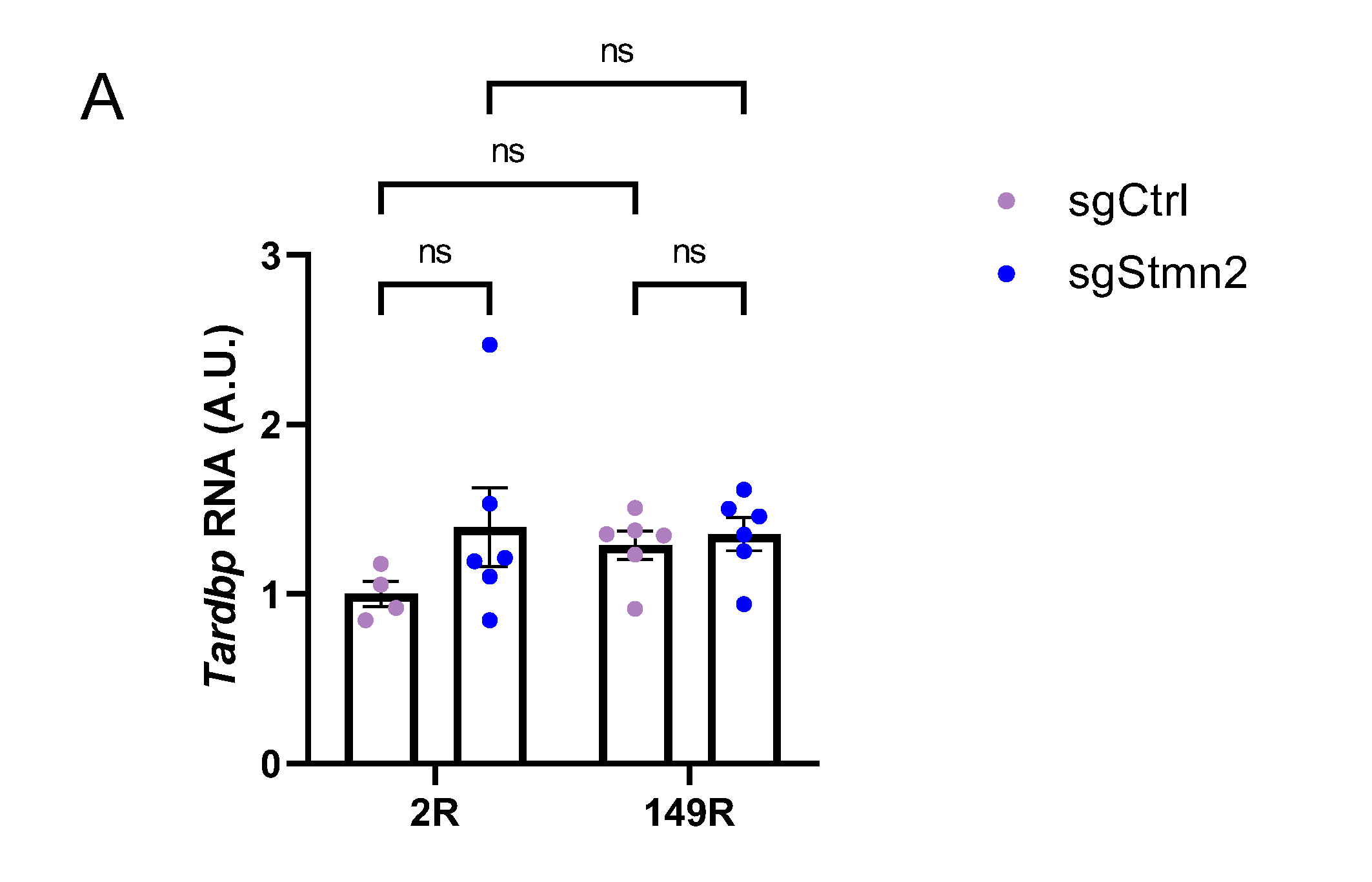

Supplement: Supplementary file 1 [file Image3.TIF]

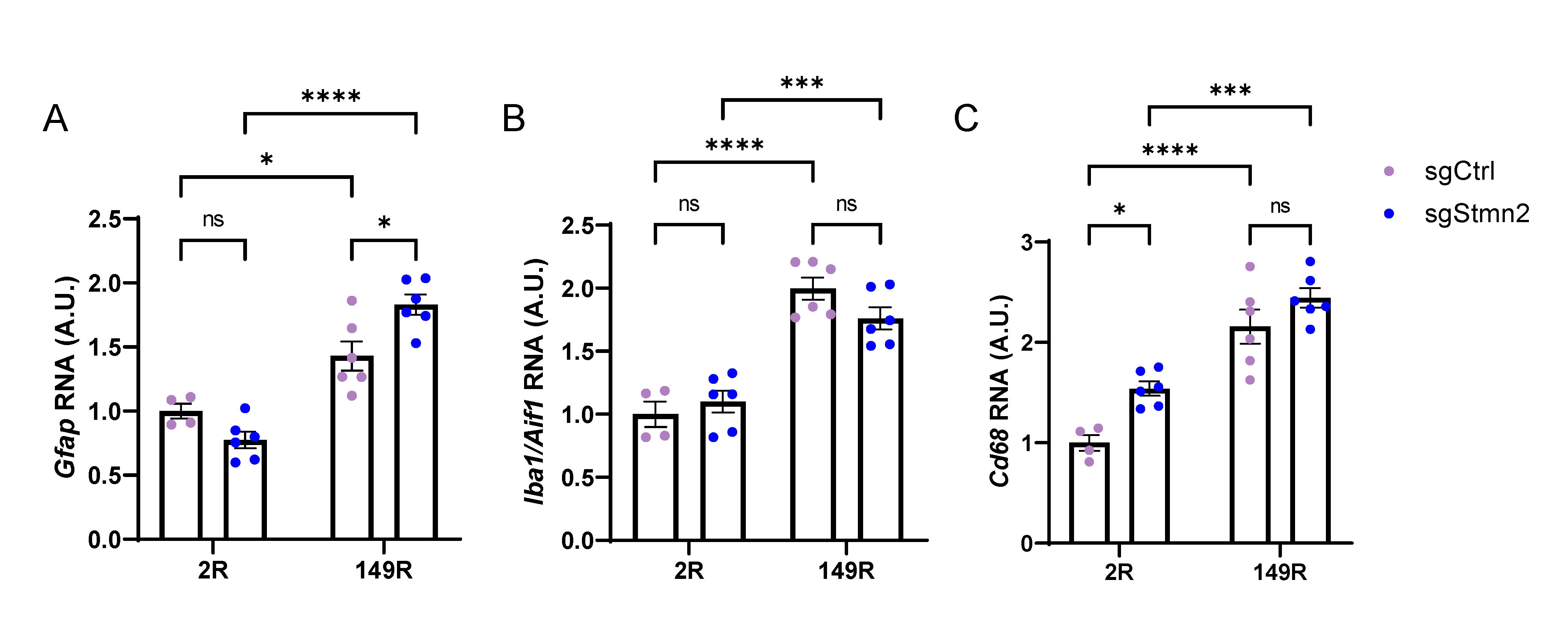

Supplement: Supplementary file 2 [file Image4.TIF]

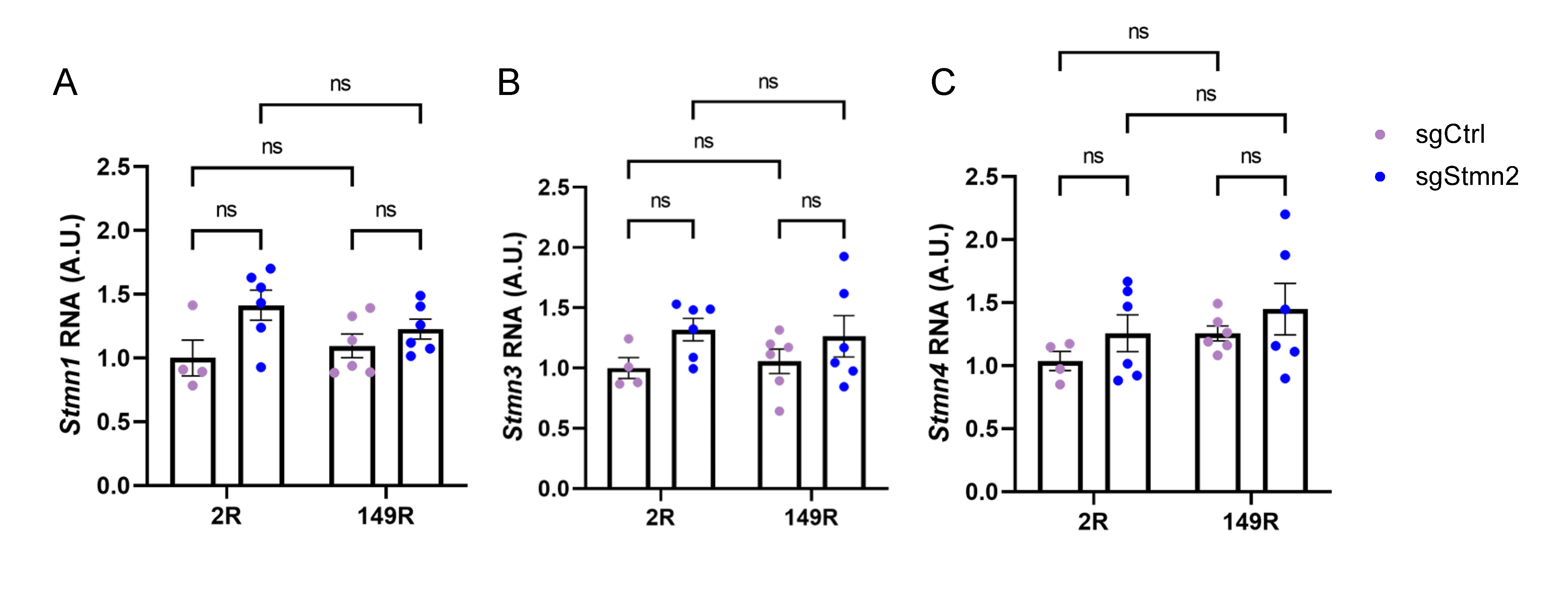

Supplement: Supplementary file 3 [file Image2.TIF]

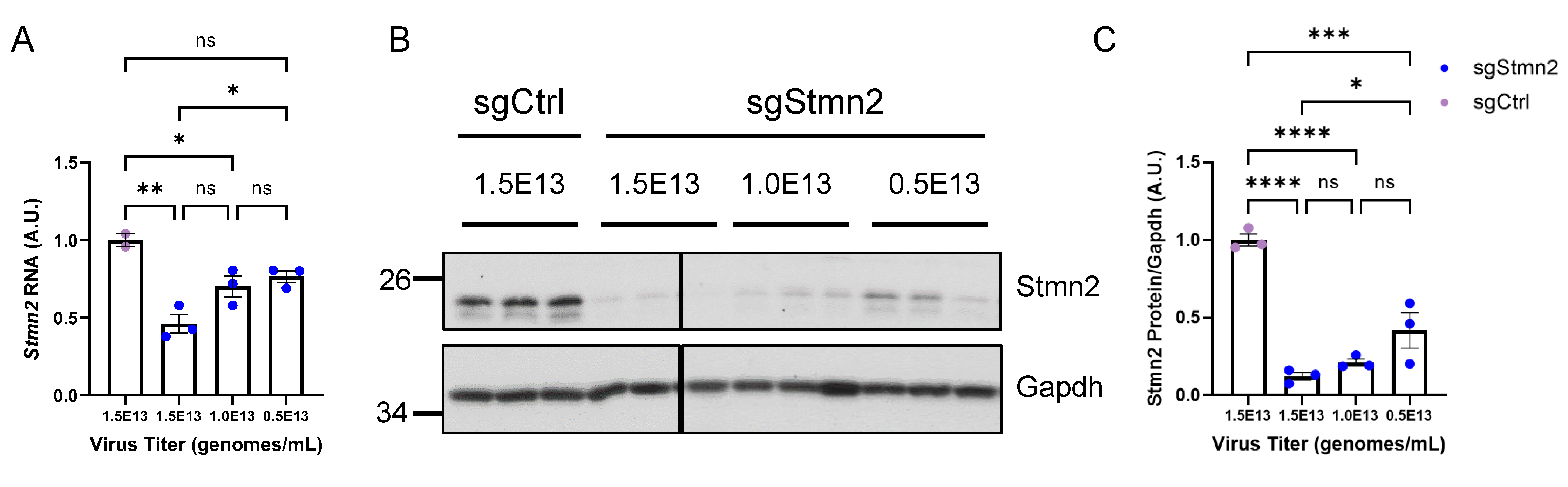

Supplement: Supplementary file 4 [file Image1.TIF]
